# Supplementary material for: Suppressing Chondrocyte Hypertrophy to Build Better Cartilage
Source: Bioengineering (Basel). 2023 Jun 20;10(6):741. doi: 10.3390/bioengineering10060741 (PMC10295138; doi:10.3390/bioengineering10060741)
Supplement: Supplementary file 1 [file bioengineering-10-00741-s001.zip › bioengineering-2430522-supplementary.pdf]

Table S1 *Ongoing Clinical Trails Aiming at Resurfacing/repairing Articular Cartilage*

ClinicalTrials.gov Identifier	Study Title	Condition	Intervention	Brief Summary	Sponsor Location	Status	Number of Participants	Primary Outcome Measure
NCT04463238	Guided Cartilage Regeneration Membrane (GCRM)	Articular Cartilage Injury	Combination Product: Guided cartilage regeneration membrane Procedure: Microfracture	Guided cartilage regeneration membrane for repairing the safety and effectiveness of articular cartilage injury.	Peking University Third Hospital - China	Not yet recruiting	150	MRI at 2 years
NCT04785092	All Autologous Cartilage Regeneration in the Treatment of the Knee Cartilage Defects	Cartilage Damage	Chondrocytes autologous transplantation (ACT) with all autologous cells	The aim of this pilot study is to evaluate the clinical performance of a modified version of the ACT technique, the All Autologous Cartilage Repair technique (AACR). It is a one-step technique in which the healthy cartilage harvested is fragmented directly in situ and then mixed with the autologous platelet concentrate and directly injected in the cartilage defect. This leads to a less invasive surgery and cost-effective procedure.	Istituto Ortopedico Rizzoli - Italy	Not yet recruiting	20	Knee functionality at baseline, 6 months, 12 months, and 24 months based on KOOS questionnaire
NCT04821102	Investigator Initiated Trial to Evaluate Cartilage Regeneration by Arthroscopy After JOINTSTEM Administration	Degenerative Arthritis Knee Arthritis	Biological: JOINTSTEM Drug: Saline	The purpose of this study is to confirm cartilage regeneration through arthroscopy after a single administration of autologous Adipose Tissue derived Mesenchymal stem cells (JOINTSTEM) in patients with degenerative arthritis of K-L grade 3.	R-Bio - Germany	Not yet recruiting	21	Arthroscopic evaluation at 48 weeks

NCT04825730	Follow-up Study for Participants of Jointstem Investigator Initiated Trial by Arthroscopy	Degenerative Arthritis Knee Arthritis	Drug: JOINTSTEM	The purpose of this study is to confirm cartilage regeneration through arthroscopy after a single administration of autologous Adipose Tissue derived Mesenchymal stem cells (JOINTSTEM) in patients with degenerative arthritis of K-L grade 3.	R-Bio - Germany	Not yet recruiting	14	Adverse events at 60 months
NCT04234412	Treatment of Osteoarthritic Knee with High Tibial Osteotomy and Implantation of Allogenic Human Umbilical Cord Blood-derived Stem Cells	Osteoarthritis, Knee	Procedure: umbilical cord blood stem cell implantation for osteoarthritis treatment	The purpose of this study is to evaluate the clinical results after implantation of human umbilical cord blood-derived mesenchymal stem cells (hUCB-MSCs) and high tibia osteotomy in patients with medial compartment osteoarthritis (MCOA) as well as varus deformity of the knee joint.	Uijeongbu St. Mary Hospital - South Korea	Not yet recruiting	10	International Cartilage Repair Society (ICRS) grade improvement Time Frame: at 24, 48, 96 weeks after surgery, score changes will be observed
NCT03219307	NOVOCART 3D Treatment Following Microfracture Failure	Articular Cartilage Injury	Combination Product: NOVOCART 3D	This study is prospective single arm extension study of protocol AAG-G-H-1220. It is open only to participants of AAG-G-H-1220 randomized to the microfracture treatment group.	Aesculap Biologics, LLC - USA	Recruiting	30	KOOS pain subdomain score Time Frame: 24 months

NCT01477008	BiPhasic Cartilage Repair Implant (BiCRI) IDE Clinical Trial	Chondral or Osteochondral Lesion of Medial Femoral Condyle, Lateral Femoral Condyle, or Trochlea	Device: BiPhasic Cartilage Repair Implant Procedure: Marrow Stimulation	The purpose of this trial is to evaluate the safety and effectiveness of the Biphasic Cartilage Repair Implant (BiCRI) compared to marrow stimulation in the treatment of chondral and osteochondral lesions located on the medial femoral condyle, lateral femoral condyle, or trochlea of the knee. The hypothesis is that the BiCRI provides an improvement in pain and function as compared to baseline, that is no worse than marrow stimulation at 1 year.	BioGend Therapeutics Co. Ltd. - Taiwan	Active, not recruiting	92	IKDC-2000 Subjective Knee Evaluation Time Frame: 12 months
NCT02673905	Clinical Trial for the Regeneration of Cartilage Lesions in the Knee (NosetoKnee2)	Tear; Knee, Cartilage, Articular	N-Cell activated Matrix (CAM) is based on autologous nasal chondrocytes expanded and further cultured on type I/III collagen membrane for 2 days vs 2 weeks	The purpose of this study is to investigate the efficacy of an engineered cartilage transplant (N-TEC) in comparison to a cell-activated matrix (N-CAM) for the treatment of articular cartilage lesions in the knee. The main innovations in this trial are the use of nasal chondrocytes and the implantation of a tissue in contrast to cells seeded on a matrix. The goals of the trial are to: (i) evaluate whether implantation of a more mature graft (tissue therapy) is beneficial for the quality and durability of the repair tissue and the clinical outcome, (ii) determine the potential of the mature graft to integrate with the adjacent cartilage and form hyaline repair tissue, and (iii) assess the efficacy of each treatment in correlation to the characteristics of the defect (e.g. "acute" versus "chronic" setting).	University Hospital, Basel, Switzerland	Active, not recruiting	108	KOOS score at 24 months

NCT02090140	Microfracture Versus Adipose Derived Stem Cells for the Treatment of Articular Cartilage Defects	Degenerative Lesion of Articular Cartilage of Knee	Procedure: ADSC Application Procedure: Microfracture	The purpose of this study is to compare two biologic methods for the treatment of articular cartilage defects in the knee. The first method, microfracture, is the standard of care and is routinely used to recruit cells from the subchondral bone marrow to the site of cartilage loss. The second method is the application of adipose-derived stem cells (ADSCs) to the defect site. In theory, ADSCs on a collagen scaffold should enable the delivery of more specific progenitor cells to the site of injury, resulting in better regeneration and integration of articular cartilage at the site of a defect as compared to the microfracture method.	University of Colorado, Denver - USA	Recruiting	17	Health Scores on the KOOS Questionnaire Time Frame: Completed at baseline, 6 months, 12 months, and 24 months post-operatively
NCT03101163	Efficacy and Safety Study of Intra-Articular Injections of Autologous Peripheral Blood Stem Cells Following Subchondral Drilling Surgery for the Treatment of Articular Cartilage Injury in the Knee	Articular Cartilage Disorder of Knee Articular Cartilage; Degeneration	Biological: Autologous peripheral blood stem cells and hyaluronic acid Other: Hyaluronic acid	This is a 24-month, multicenter, randomized, open-label, standard treatment-controlled, parallel-group, Phase 2 study for adults with large or complicated knee articular cartilage lesions and are candidates for knee joint cartilage repair surgery. The safety and efficacy of intra-articular injections of peripheral blood stem cells (PBSCs) together with hyaluronic acid (HA) after subchondral drilling surgery will be evaluated to determine whether PBSC therapy can improve functional outcome and reduce pain of the knee joint better than a standard treatment (HA injections and physiotherapy regimen).	KLSMC Stem Cells, Inc. - USA	Active, not recruiting	120	IKDC score and KOOS score at 24 months

NCT03321812	Clinical Study of Decalcification Bone Scaffold for Cartilage Lesions of the Knee	Cartilage Injury	Procedure: decalcification bone scaffold Procedure: microfracture	The trial evaluates the clinical efficacy and safety of decalcification bone scaffold for cartilage lesions of the knee. Half of participants will receive decalcification bone scaffold combined with microfracture, while the other will only receive microfracture as a control group.	Peking University Third Hospital - China	Recruiting	60	Appraise of magnetic resonance imaging Time Frame: 5 years
NCT02659215	HyaloFAST Trial for Repair of Articular Cartilage in the Knee (FastTRACK)	Defect of Articular Cartilage	Device: Hyalofast Procedure: Microfracture	The purpose of this study is to evaluate the safety and efficacy of Hyalofast® scaffold with bone marrow aspirate concentrate (BMAC) compared to microfracture in the treatment of symptomatic cartilage defects of the knee.	Anika Therapeutics, Inc. - Italy	Recruiting	200	IKDC score and KOOS score at 24 months
NCT03801564	Knee Osteoarthritis: Platelet Rich Plasma or Hyaluronic Acid	Knee Osteoarthritis	Device: Platelet Rich Plasma Treatment Group (PRP) Device: Hyaluronic Acid Treatment Group (HA)	The purpose of this study is to compare the effect of hyaluronic acid or platelet-rich plasma on pain, physical function, quality of life and knee joint morphology in patients with knee osteoarthritis severity II-III.	Antalya Training and Research Hospital - Turkey	Enrolling by invitation	120	Change from Baseline Health Related Quality of Life Measured by Western Ontario and McMaster Universities Osteoarthritis Index (WOMAC) Periodically during 12 months Time Frame: The scale is filled before the first and second injection which is one month apart, then the evaluation continues at 3rd, 6th, 9th, and 12th months

NCT04187183	Use of Fresh Platelet Rich Plasma with Concentrated Leukocytes or Fresh Platelet Rich Plasma Without Concentrated Leukocytes in the Treatment of Knee Cartilage Degeneration: a Randomized Controlled Trial	Osteoarthritis, Knee	Other: Fresh Platelet Rich Plasma with leukocyte Other: Fresh Platelet Rich Plasma without leukocyte	The aim of the study is to compare the triple infiltration of Fresh Platelet Rich Plasma with concentrated Leukocytes against triple infiltration of Fresh Platelet Rich Plasma Without Concentrated Leukocytes in the treatment of Knee Cartilage Degeneration in a Double Blind Randomized Controlled Trial.	Istituto Ortopedico Rizzoli - Italy	Recruiting	132	IKDC-subjective score (International Knee Documentation Committee) Time Frame: 12 months
NCT03790189	Subchondral and Intra-articular Application of Bone Marrow Concentrate for Knee Uni-compartmental OA (Marrow Mule)	Osteoarthritis, Knee Cartilage Degeneration	Biological: Subchondral and intra-articular injection of BMC	The aim of the present pilot trial is to assess the safety and describe the clinical outcome following concurrent intra-articular and subchondral bone application of Bone Marrow Concentrate (BMC). BMC is an autologous product rich in mesenchymal stem cells, which have immuno-modulatory and trophic properties and are able to restore the joint homeostasis by reducing the inflammatory distress traditionally associated to osteoarthritis.	Istituto Clinico Humanitas - Italy	Recruiting	25	Change in IKDC (International Knee Documentation Committee) subjective score Time Frame: baseline vs 12 months follow-up
NCT04186208	Non-interventional Study With NOVOCART® 3D for the Treatment of Cartilage Defects of the Knee in Pediatric Patients (JUNOVO)	Cartilage Disease	Drug: NOVOCART 3D	Prospective, multicenter, single-arm non-interventional study to evaluate the efficacy, safety and health economics of NOVOCART 3D in the treatment of cartilage defects of the knee in pediatric patients with closed epiphyseal growth plates.	Tetec AG - Germany	Recruiting	40	Change of Overall KOOS (Knee Injury and Osteoarthritis Outcome Score) Time Frame: 24 months follow-up

NCT04236739	Comparing Clinical Outcomes of the One-step Cartilage Transplantation in Cartilage Defects of the Knee with Conservative Treatment (IMPACT2)	Cartilage Damage	Drug: Instant MSC Product accompanying Autologous Chondron Transplantation	The objective of IMPACT2 is to compare clinical outcomes of 30 individual participants with cartilage defects treated with IMPACT to 30 participants treated with standard care for 9 months (consisting of optional physical therapy and pain medication).	R.J.H. Custers - Netherlands	Recruiting	60	KOOS change on scale of 0-100<br>Time Frame: At baseline, 3, 6 and 9 months
NCT03873545	Prospective Evaluation of ProChondrix CR for Repair of Articular Cartilage Defects on Femoral Condyle and Patella	Cartilage Injury<br>Cartilage Damage	Procedure: Cryopreserved Osteochondral Allograft	The objective is to evaluate the use of ProChondrix Cryopreserved Osteochondral Allograft to obtain evidence of effectiveness, defined as an improvement in physical function and pain, when used on a symptomatic cartilage defect on the femoral condyle or patella in a mechanically stable knee.	AlloSource - USA	Recruiting	80	Subjective International Knee Documentation Committee (IKDC) Score<br>Time Frame: Baseline, 3, 6, 12, 24, 36, 48 and 60 months
NCT03672825	REcycled CartiLage Auto/Allo IMplantation	Cartilage Defect	Drug: REcycled CartiLage Auto/Allo IMplantation	This is an FDA phase I study to evaluate the safety of allogeneic culture-expanded adipose-derived mesenchymal stem cells (AMSCs) combined with autologous cartilage cells to treat focal knee cartilage defects in one stage surgery.	Mayo Clinic - USA	Recruiting	25	Nature, incidence, and severity of adverse events<br>Time Frame: 2 years
NCT03072147	Teriparatide as a Chondro-regenerative Therapy in OA	Knee Osteoarthritis<br>Cartilage Degeneration	Drug: Teriparatide<br>Drug: Placebo	The purpose of this study is to evaluate teriparatide (TP) as a chondroregenerative therapy for human knee osteoarthritis (OA). The central hypothesis to be tested is that TP supports structural modification of the joint and improves biomarker, functional and patient-reported measures of knee OA.	University of Rochester - USA	Active, not recruiting	76	Knee MRI<br>Time Frame: Change from Baseline through study completion (baseline, 24 weeks, 48 weeks), an average of one year

NCT04814368	A Safety and Efficacy Study of Anti-inflammatory (Canakinumab) and Cartilage Stimulating (LNA043) Drugs Injected into the Knee Joint of Participants with Knee Osteoarthritis (OA)	Knee Osteoarthritis	Biological: canakinumab Biological: LNA043Other: Placebo to canakinumab	The study will establish safety and efficacy of canakinumab and LNA043 in patients with knee osteoarthritis (OA).	Novartis Pharmaceuticals - USA	Not yet recruiting	138	Change in cartilage volume in the index region measured by MRI Time Frame: Baseline to Day 197
NCT04210986	Senolytic Drugs Attenuate Osteoarthritis-Related Articular Cartilage Degeneration: A Clinical Trial	Osteoarthritis, Knee	Dietary Supplement: Fisetin Drug: Placebo oral capsule	Phase I/II randomized, double-blind, placebo-controlled clinical trial to test the safety and efficacy of Fisetin for treating mild to moderate osteoarthritis.	Steadman Philippon Research Institute - USA	Recruiting	72	Incidence of Treatment-Emergent Adverse Events as assessed by blood chemistries (Liver, Kidney, Lysis Syndrome) Time Frame: Duration of study, an average of 12 months
NCT04097379	Safety, Tolerability and Preliminary Efficacy of Multiple Intra-articular Injections of LRX712 in Patients with Knee OA	Osteoarthritis (OA)	Drug: LRX712Drug: Placebo	This study will explore the preliminary efficacy of multiple intra-articular injections of LRX712 by evaluating the ability of the drug to restore structural integrity of articular cartilage. Efficacy will be evaluated in the context of the systemic safety and local tolerability of the investigational drug.	Novartis Pharmaceuticals - Netherlands	Recruiting	40	Changes in articular cartilage [23Na] content from baseline compared to placebo at week 28 Time Frame: Baseline and Week 28
